# American Dentists in Europe

**Published:** 1858-10

**Authors:** 


*American Dentists in Europe.*—There are, we believe, from twelve to eighteen American dentists engaged in practice at the present time in different parts of Europe, and all, so far as we have been able to learn, are receiving the most flattering encouragement. There are two in London, one in Manchester, three or four in Paris, one in Berlin, one in Rome and one in Madrid. There are also others whose places of residence we do not know, and every year adds to the number. We have recently learned that Dr. F. Fuller of Portsmouth, N. H., intends visiting Europe in a few months, but whether he goes merely for purposes of pleasure or with a view of practicing his profession abroad, we are not informed. A gentleman of his intelligence and professional capacity will scarcely fail to meet with a kind reception from the members of his profession on the other side of the Atlantic. We wish him a pleasant trip and safe return.

